# Differential co‐expression network analysis elucidated genes associated with sensitivity to farnesyltransferase inhibitor and prognosis of acute myeloid leukemia

**DOI:** 10.1002/cam4.6804

**Published:** 2023-12-08

**Authors:** Nurdan Kelesoglu, Medi Kori, Betul Karademir Yilmaz, Ozlem Ates Duru, Kazim Yalcin Arga

**Affiliations:** ^1^ Department of Bioengineering Marmara University Istanbul Türkiye; ^2^ Genetic and Metabolic Diseases Research and Investigation Center Marmara University Istanbul Türkiye; ^3^ Department of Biochemistry, Faculty of Medicine Marmara University Istanbul Türkiye; ^4^ Department of Nutrition and Dietetics, School of Health Sciences Nişantaşı University Istanbul Türkiye; ^5^ Department of Chemical Engineering, Faculty of Engineering Bolu Abant İzzet Baysal University Bolu Türkiye

**Keywords:** acute myeloid leukemia, biomarkers, network biomedicine, therapeutics, transcriptomics

## Abstract

Acute myeloid leukemia (AML) is a heterogeneous disease and the most common form of acute leukemia with a poor prognosis. Due to its complexity, the disease requires the identification of biomarkers for reliable prognosis. To identify potential disease genes that regulate patient prognosis, we used differential co‐expression network analysis and transcriptomics data from relapsed, refractory, and previously untreated AML patients based on their response to treatment in the present study. In addition, we combined functional genomics and transcriptomics data to identify novel and therapeutically potential systems biomarkers for patients who do or do not respond to treatment. As a result, we constructed co‐expression networks for response and non‐response cases and identified a highly interconnected group of genes consisting of SECISBP2L, MAN1A2, PRPF31, VASP, and SNAPC1 in the response network and a group consisting of PHTF2, SLC11A2, PDLIM5, OTUB1, and KLRD1 in the non‐response network, both of which showed high prognostic performance with hazard ratios of 4.12 and 3.66, respectively. Remarkably, ETS1, GATA2, AR, YBX1, and FOXP3 were found to be important transcription factors in both networks. The prognostic indicators reported here could be considered as a resource for identifying tumorigenesis and chemoresistance to farnesyltransferase inhibitor. They could help identify important research directions for the development of new prognostic and therapeutic techniques for AML.

**Abbreviations**
AML: Acute myeloid leukemia
DEG: differentially expressed gene
GEO: Gene Expression Omnibus
HTRdb: Human Transcriptional Regulation Interactions database
KM: Kaplan–Meier
LIMMA: Linear Models for Microarray Data
TF: transcription factor

## 1 INTRODUCTION

Acute myeloid leukemia (AML) is defined by the accumulation of several acquired genetic abnormalities in hematopoietic progenitor cells, resulting in impaired cell proliferation and differentiation.^1^ AML is a dynamic disease that cannot be effectively categorized into predefined groups since most patients with AML co‐express multiple different mutations.^2^ Despite significant advances in the comprehension of AML pathogenesis, the general prognosis for AML patients remains poor, with a considerable proportion of patients dying from disease relapse or failing to respond to treatment. A better knowledge of the disease mechanism that causes AML might contribute to developing more targeted and less harmful treatments for the disease.

Tipifarnib, an inhibitor of farnesyltransferase, acts by inhibiting the binding of Ras to the membrane, resulting in its deactivation. This mechanism has demonstrated the ability to inhibit proliferation of various human tumor cell lines in *in vitro* and *in vivo* studies.^3^, ^4^ In a phase 1 clinical trial, tipifarnib demonstrated a 32% response rate in patients with AML.^5^ Through microarray analysis, researchers identified eight gene markers that can predict response to tipifarnib.^6^ Among these markers, a subset showed not only predictive value for drug response, but also potential significance in the field of farnesyltransferase inhibitor biology. The study examined the potential of tipifarnib in AML treatment and showed that gene expression analysis of bone marrow samples from a phase 2 clinical trial revealed tipifarnib‐induced changes in gene profiles associated with cellular processes, with a subset of 27 genes showing differential modulation between responders and non‐responders, potentially serving as predictive biomarkers of treatment efficacy.^7^

The expanding access to high data from AML patients' bone marrow and blood presents remarkable prospects for thoroughly identifying AML diagnostic biomarkers that could augment and update current ones. Differential co‐expression is used to associate the differences of unknown functions of genes between two groups, evaluate potential disease genes, and identify how to function in the prognosis.^8^ In contrast to differential co‐expression analyses, which assess correlations among gene expression patterns, omics analyses do not examine the possibility of cooperation among differentially expressed genes (DEGs). To gain a systems‐level knowledge of genome reprogramming under disease processes, differential gene expression analysis should be supported with differential co‐expression analyses.^9^, ^10^, ^11^

While previous studies focused on specific gene expression assays such as RASGRP1/APTX gene expression ratio^12^ and AKAP13 gene expression signature^6^ to categorize patients or predict their response to tipifarnib, here we employed an integrative network analysis pipeline and further evaluated the response to farnesyltransferase inhibitor (tipifarnib) treatment in patients with AML to examine differentially co‐expressed networks. Integrative network analysis allowed us to identify interconnected gene modules and regulatory pathways that may collectively influence response to tipifarnib in AML patients. This comparative analysis led us to propose a collection of hub genes with potential implications as prospective biomarkers for adult AML patients. These biomarkers could increase the accuracy of predicting response to treatment and help physicians make more informed decisions about personalized therapeutic strategies for AML patients. Moreover, by introducing this integrative network analysis pipeline, we hope to improve the current understanding of how AML patients respond to tipifarnib. Insights gained from this study could contribute to the development of more targeted and effective treatments that will ultimately improve patient outcomes and bring us one step closer to realizing precision medicine approaches for the treatment of AML.

This study aims to contribute to this growing area of research by exploring differential co‐expression analysis to identify altered gene co‐expression patterns between two phenotypes, such as treatment response versus nonresponse patient data. Notably, differently co‐expressed gene networks can reveal details about disease development, progression, prognosis, and regulatory processes. Our study assesses the significance of the networks in the responded/non‐responded states, makes an integrative network analysis pipeline, compares these differently co‐expressed networks, and recommends a collection of hub genes as prospective biomarkers in AML.

## 2 MATERIALS AND METHODS

### 2.1 Transcriptome datasets

Deciphering individual differences in disease development and treatment response was the goal of the current differential co‐expression analysis. The inclusion criteria were datasets relevant to these goals, datasets with adult patient groups, and datasets with at least 25 samples each (Table 1).

**TABLE 1:** Transcriptome datasets employed in the current study.

Source‐ID	Disease	Purpose	# of samples	References
GEO‐GSE5122	Acute myeloid leukemia	Network identification	53 (9 responded, 44 unresponded) (Relapse and Refractory AML patients)	^6^
GEO‐GSE8970	Acute myeloid leukemia	Network identification	26 (13 responded, 13 unresponded) (Previously untreated AML patients, comprised of de novo and secondary AML patients)	^12^
TCGA‐LAML	Acute myeloid leukemia	Prognostic performance	200 (untreated)	^16^
TCGA‐DLBC	Diffuse large B‐cell lymphoma	Prognostic performance	58 (untreated)	^17^
TCGA‐CLL	Chronic lymphocytic leukemia	Prognostic performance	201 (untreated)	^18^

To prevent microarray platform differences, the datasets obtained from the same microarrays and Affymetrix microarray platforms were employed. Two transcriptome datasets (GSE5122, and GSE8970) were downloaded from the Gene Expression Omnibus (GEO)^13^ database.

The individuals in the GSE5122^6^ dataset were relapsed or refractory AML patients. Within this cohort, 27 patients were classified as refractory, while 31 patients were categorized as relapsed. GSE8970,^12^ consists of previously untreated as nine de novo AML and 25 secondary AML patients. The samples in both datasets were collected from patients before treatment. The starting dose of tipifarnib treatment was 600 mg administered orally twice daily for 21 consecutive days in 4‐week cycles for patients with refractory or relapsed AML. Therapy could continue until disease progression or unacceptable toxicity.^14^ Previously untreated patients received tipifarnib twice daily for 21 days, followed by a rest period of up to 42 days to allow their blood counts to recover.^15^ If their condition improved or stabilized, they could receive additional cycles of therapy. In complete remission, up to 4 cycles were possible, while in partial remission, treatment could continue as long as there were no adverse effects or disease progression. To discover gene signatures linked to treatment response, both datasets were employed in the comparative analysis of treatment response groups, i.e., responders and non‐responders. GSE5122, consisting of 58 patients. Thirty one individuals had specific cytogenetic risk profiles, such as abnormal chromosome 5, 7, 11 translocations, inversion of chromosome 16, and others, while the remaining patients had a normal karyotype.^14^ GSE8970 included patients classified as poor risk with unfavorable cytogenetic profiles, including abnormalities such as −5/5q, −7/7q, +8, abn 11q, and complex karyotypes that have three or more unrelated abnormalities.^15^

The Cancer Genome Atlas (TCGA) provided independent RNA‐Seq datasets that included clinical information about patients and were used in prognostic analyses (Table 1).^16^, ^17^, ^18^ Patients with a complete or hematologic response were classified as “Treatment response” in both datasets. On the other hand, “Stable disease” was defined as no hematological response but no disease progression.^6^ Patients with stable disease were not included in the analysis because they neither responded nor did not respond to the study target. Five of the total 58 patients were excluded from GSE5122 because they did not meet our criterion for treatment response. In the dataset, response was determined, with 9 patients showing a positive response to treatment and 44 patients not responding positively. GSE8970 originally enrolled 34 patients, but eight were excluded based on their response. Among them, 13 patients showed a positive response to treatment, while the remaining 13 patients did not respond to treatment. After preprocessing of the data in the GSE5122 and GSE8970 datasets, the male‐to‐female ratio was 26:27 and 18:8, respectively, and the mean age was 60 and 73 years, respectively.

Tipifarnib, a farnesyltransferase inhibitor, was used in the treatment of patients in both datasets.

### 2.2 Differential gene expression analysis

The DEGs were characterized using a previously developed statistical analysis method.^19^ Each dataset was normalized by the Robust Multi‐Array Average^20^ as implemented in the “affy” package^21^ of the R/Bioconductor platform.^22^ Multiple testing options of LIMMA^23^ were used to determine DEGs from normalized log expression values. Benjamini‐ Hochberg's method was used to control for false discovery rate. An adjusted *p*‐value threshold of 0.05 and fold change cutoff of 1.5 were used to determine the statistical significance and the direction of regulation (i.e., up‐ and down‐regulated genes).

### 2.3 Correlated gene pairs in response status

Pearson's correlation coefficients (PCCs) were used to calculate the correlation patterns of each core gene pair in response status as responders and non‐responders. In the co‐expression analysis, we used the threshold value of 0.7^24^, ^25^, ^26^ which is generally accepted as a significant PCC level. When the associated PCC is greater than 0.7, the gene pair was considered positively co‐expressed, and the PCC between genes less than −0.7 was considered negatively co‐expressed.

To identify differential co‐expression profiles between two conditions, the following formula was applied:

$$|\left({\mathrm{PCC}}_{\text{Response}}-{\mathrm{PCC}}_{\text{Nonresponse}}\right)/{\mathrm{PCC}}_{\text{Response}}|\geq 1$$

where ${\mathrm{PCC}}_{\text{Response}}$ and ${\mathrm{PCC}}_{\text{Nonresponse}}$ are the PCCs of DEG pair in response and nonresponse states, respectively. Gene pairs with significant co‐expression patterns in any of the phenotypes were included in the differential co‐expression analysis.

### 2.4 Construction of co‐expression networks based on response status

To construct differential gene co‐expression networks around differentially co‐expressed gene pairs, four different conditions were considered; (1) gene pairs with positive correlations in the responded state but no correlation in the nonresponded state (PO), (2) gene pairs with negative correlations in the responded state but no correlation in the non‐responded state (NO), (3) gene pairs with no correlations in the responded state but with positive correlation in the non‐responded state (OP), (4) gene pairs with no correlations in the responded state but with negative correlation in the non‐responded state (ON). Two co‐expression networks (PONO and OPON) were constructed within the significantly co‐expressed DEGs. PONO shows the co‐expression networks of responded state, whereas OPON represents correlations in the nonresponded state. Functional enrichment analyses were performed for the gene sets in the networks using the ConsensusPathDB database.^27^ An adjusted p < 0.05 was used to describe statistical significance.

### 2.5 Determination of differential co‐expression network modules

Cytoscape software^28^ (v3.9) and Cytohubba plug‐in^29^ were used to visualize the differential gene co‐expression networks and determine hub genes. Degree, as a local metric, and betweenness centrality, as a global metric, were used to characterize hub genes. The highly connected hubs (in terms of both degree and betweenness centrality metrics) that form an integral part of the network were accepted as a module.

### 2.6 Identification of transcriptional regulatory networks

Experimentally validated TF–target gene interactions from the current version of the Human Transcriptional Regulation Interactions database (HTRIdb)^30^ and the combinatorial human transcriptional regulatory interaction network^31^ were used to analyze the connection of TFs with network genes. TFs that regulated the genes were presented in the analysis for each significant network.

### 2.7 in silico validation and prognostic performance

The genes of differentially co‐expressed networks were investigated in AML and two different tumor types (Chronic lymphocytic leukemia‐CLL and Diffuse large B‐cell lymphoma‐DLBCL) to determine the specificity of the response and nonresponse networks of AML and to analyze the expression pattern of the network in the different hematological malignancies. Cox proportional hazards regression analysis in the SurvExpress^32^ validation tool and RNA‐Seq Datasets obtained from TCGA were used to identify prognostic capabilities of the genes in the differentially co‐expressed networks. The samples were divided into low‐ and high‐risk categories in the SurvExpress based on their prognostic index. Kaplan–Meier graphs, log‐rank test p‐value, and the hazard ratio were used to obtain the prognostic performance of the networks.

### 2.8 The evaluation of the immune microenvironment of the networks in AML

Patient prognosis and treatment response were associated with the presence of tumor‐infiltrating leukocytes. Immunohistochemistry and flow cytometry can be difficult to implement and standardize due to their limited phenotypic markers. To gain better insight into the respective protein levels, the expression levels of network genes in immune cells were determined using the CIBERSORT tools.^33^ This method provided a visual comparison of the network landscape of immune cells.

## 3 RESULTS

### 3.1 Identification of differentially expressed genes in AML

Gene expression profiles and associated clinical data from two datasets (GSE5122 and GSE8970) were retrieved from the GEO database. Patients with relapsed and refractory AML were included in the GSE5122 dataset, whereas patients with previously untreated AML were included in the GSE8970 dataset. All AML patients were categorized into response/nonresponse status, and up‐ and down‐regulated DEGs were identified by adjusted p‐value <0.05 and fold change >1.5 (for up‐regulation) or <0.5 (for down‐regulation). DEGs were identified as 141 up‐ and 281 down‐regulated genes in GSE5122 and 1351 up‐regulated and 881 down‐regulated genes in GSE8970. Between the two datasets, 82 mutual DEGs were identified.

### 3.2 Detection and functional association of differential gene co‐expression profiles in AML

To define the biological activities, enrichment analysis was performed for up and down DEGs. The up‐regulated genes significantly enriched in MAPK Signaling Pathway, RHOH GTPase cycle, immunoregulatory interactions between a lymphoid and a non‐lymphoid cell, response to an inorganic substance, leukocyte mediated immunity, and regulation of immune system process (Figure S1A). In addition, membrane trafficking, TCR signaling, neutrophil degranulation, peptide hormone metabolism, tube development, embryonic morphogenesis, and response to nerve growth factors are conspicuous in the enrichment analysis of downregulated DEGs (Figure S1B).

**FIGURE 1:**
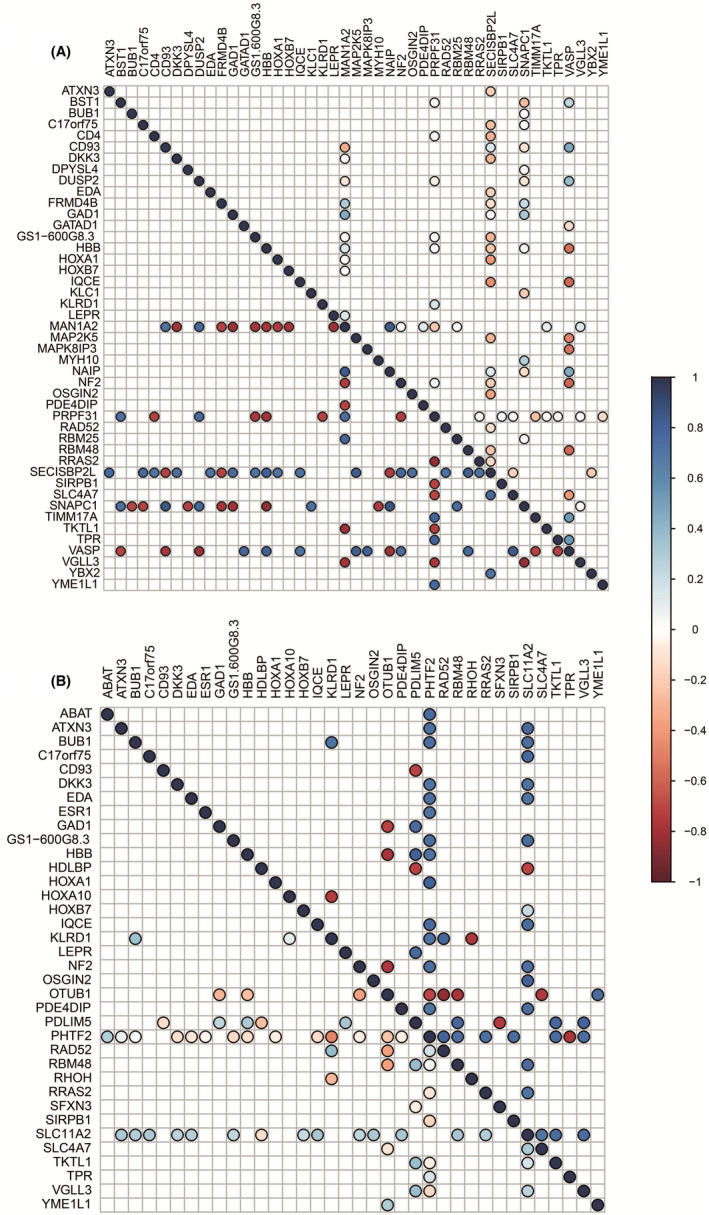
The correlation plots of hub genes were determined as modules. In the correlation plots, the lower triangle represents the co‐expression patterns in response state whereas the upper triangle represents the co‐expression patterns of genes in nonresponse state. (A) PONO (B) OPON.

The possible co‐expression profiles of 82 common DEGs were analyzed with PCC in response and nonresponse status. Four differential gene co‐expression networks were created around the differentially co‐expressed gene pairs. To reveal the co‐expression networks in the responded sss state, the gene pairs with positive correlations (PO) were combined with gene pairs with negative correlations (NO). In contrast, the co‐expression network of the nonresponsive state was analyzed by combining gene pairs with positive correlation (OP) and negative correlation (ON). The response network (PONO) included 51 genes and 88 correlations, whereas the nonresponse network (OPON) included 65 connections between 43 genes. To define the biological activities, enrichment analysis was performed for up and down DEGs. Because we are searching for a prognostic gene module that could represent signatures for treatment response in AML, we focused on the hub genes of the networks and determined them to be modules (Figure 1). Moreover, we assume that the potential prognostic modules include a substantial number of genes and a high degree of connectivity within the module to obtain high precision in their predictive ability. MAN1A2, SNAPC1, PRPF31, SECIS2BPL, and VASP were found to be highly connected hub genes in the PONO networks (Figure S2). The prominent hub genes in the OPON network were PHTF2, SLC11A2, PDLIM5, OTUB1, and KLRD1 (Figure S3).

It is important to keep in mind that this study used both datasets, which contained only patient data and no healthy samples; therefore, some genes were common in both networks. Interestingly, GAD1, NF2, HBB, and SLC4A7 appeared in four (PO‐NO‐OP‐ON) differential gene co‐expression networks. These genes are closely associated with hub genes in each network. A high proportion of PONO and OPON network genes were prognostic markers in renal and liver cancer.^34^, ^35^ In addition, six genes of the response and five genes of the nonresponse network were prognostically associated as biomarkers in endometrial cancer.

### 3.3 Transcriptional Regulators of network genes

We performed sequential evaluations to identify the critical transcriptional factors regulators of the networks to elucidate the mechanism in the differential co‐expression pattern of drug response. A total of 17 TFs were found in the PONO networks, and the six TFs (ETS1, GATA2, AR, YBX1, FOXP3, and PRDM14) regulated the majority of genes in the network. A similar result was seen in the OPON networks; the first five TFs were the same. GATA1 was found instead of PRDM14 in the PONO network. In addition, ETS1, GATA2, and AR co‐regulated 27 genes in the PONO network, whereas 25 genes in the OPON network were co‐regulated by the same TFs.

### 3.4 Prognostic performance of response and nonresponse networks

**FIGURE 2:**
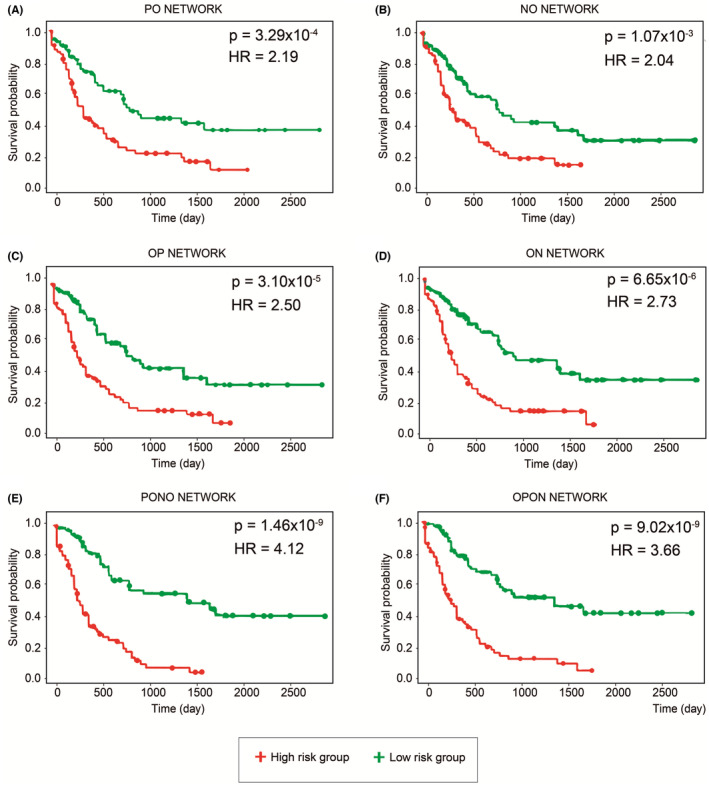
Prognostic power of hub related module genes when analyzed separately. (A) PO, (B) NO, (C) OP, (D) ON and together (E) PONO, (F) OPON. The samples were partitioned into low‐ and high‐risk groups according to their prognostic index. Kaplan Meier plot of co‐expressed genes was drawn. The *p*‐values were calculated via the log‐rank test (*p* < 0.05).

Cox proportional hazards regression analysis was performed to evaluate the prognostic performance of response and nonresponse AML networks. A comprehensive RNA‐seq dataset was used in the studies, and samples were classified into low‐ and high‐risk groups based on their prognostic index. Kaplan–Meier plots and long‐rank test were used. The prognostic performance of the PO (positively regulated) and NO (negatively regulated) response network was analyzed, and the hazard ratios were estimated as 2.19 (*p* = 3.20 × 10^−4^) and 2.04 (*p* = 0.001), respectively (Figure 2A,B). Moreover, prognostic analysis for the OP (positively regulated) and ON (negatively regulated) nonresponse network showed that the hazard ratio was estimated to be 2.5 (*p* = 3.1 × 10^−5^) and 2.73 (*p* = 6.65 × 10^−6^), respectively (Figure 2C,D).

Conversely, prognostic performance was higher when the response network was analyzed together (positive and negative regulation) than when it was analyzed alone. Similar results were obtained for the nonresponse network. The analysis showed the high performance of PONO (HR =4.12, *p* = 1.45 × 10^−9^) and OPON (HR =3.66, *p* = 9.02 × 10^−9^) networks as prognostic network biomarkers (Figure 2E,F). Since they are not independent networks, understanding their interaction may shed light on their regulation. It may provide clues to the right therapy for the disease by understanding what kind of mechanism it has in different treatment techniques and during treatment.

**FIGURE 3:**
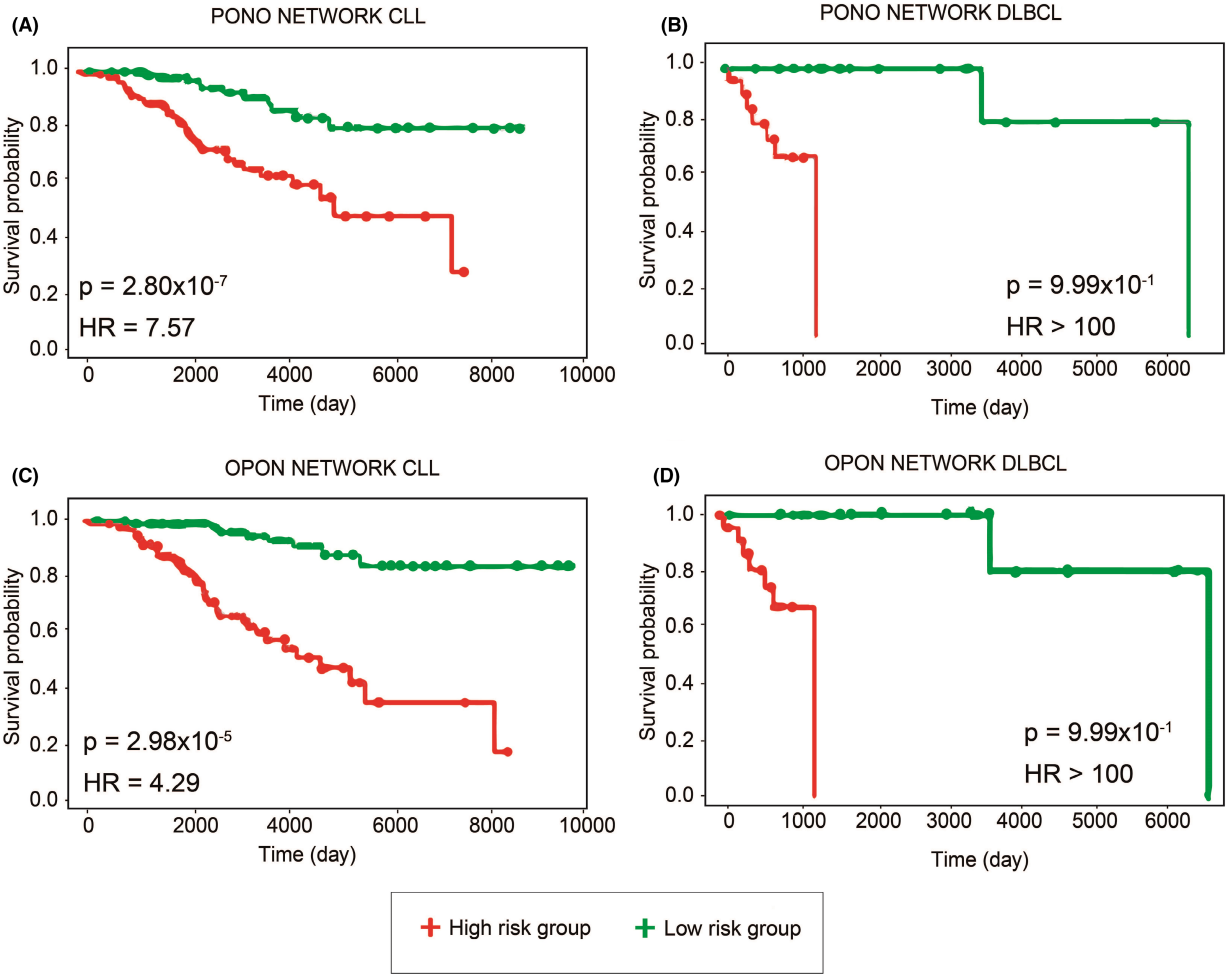
Prognostic power of hub related module genes in two types of cancer. Cox survival analysis was performed of hub genes using two types of hematological cancer. Hub genes of PONO were shown in (A) Chronic lymphocytic leukemia, (B) Diffuse large B‐cell lymphoma and PONO in (C) Chronic lymphocytic leukemia and (D) Diffuse large B‐cell lymphoma.

Differentially co‐expressed networks were studied in two different tumor types (CLL and DLBCL) types to determine the specificity of response and nonresponse networks in AML and to analyze the expression patterns of the networks in the different hematological malignancies. The response network (PONO) and nonresponse network (OPON) showed high prognostic performance in CLL (HR =7.57, *p* = 2.8 × 10^−7^ and HR =4.29, *p* = 2.9 × 10^−5^, respectively), while both PONO and OPON networks showed insignificant performance in DLBCL (*p* = 0.99 for PONO and *p* = 0.99 for OPON) (Figure 3). This result indicates that both networks can be considered prognostic for myeloid and lymphocytic leukemia, but not for DLBCL.

### 3.5 Immune microenvironment analysis of networks

**FIGURE 4:**
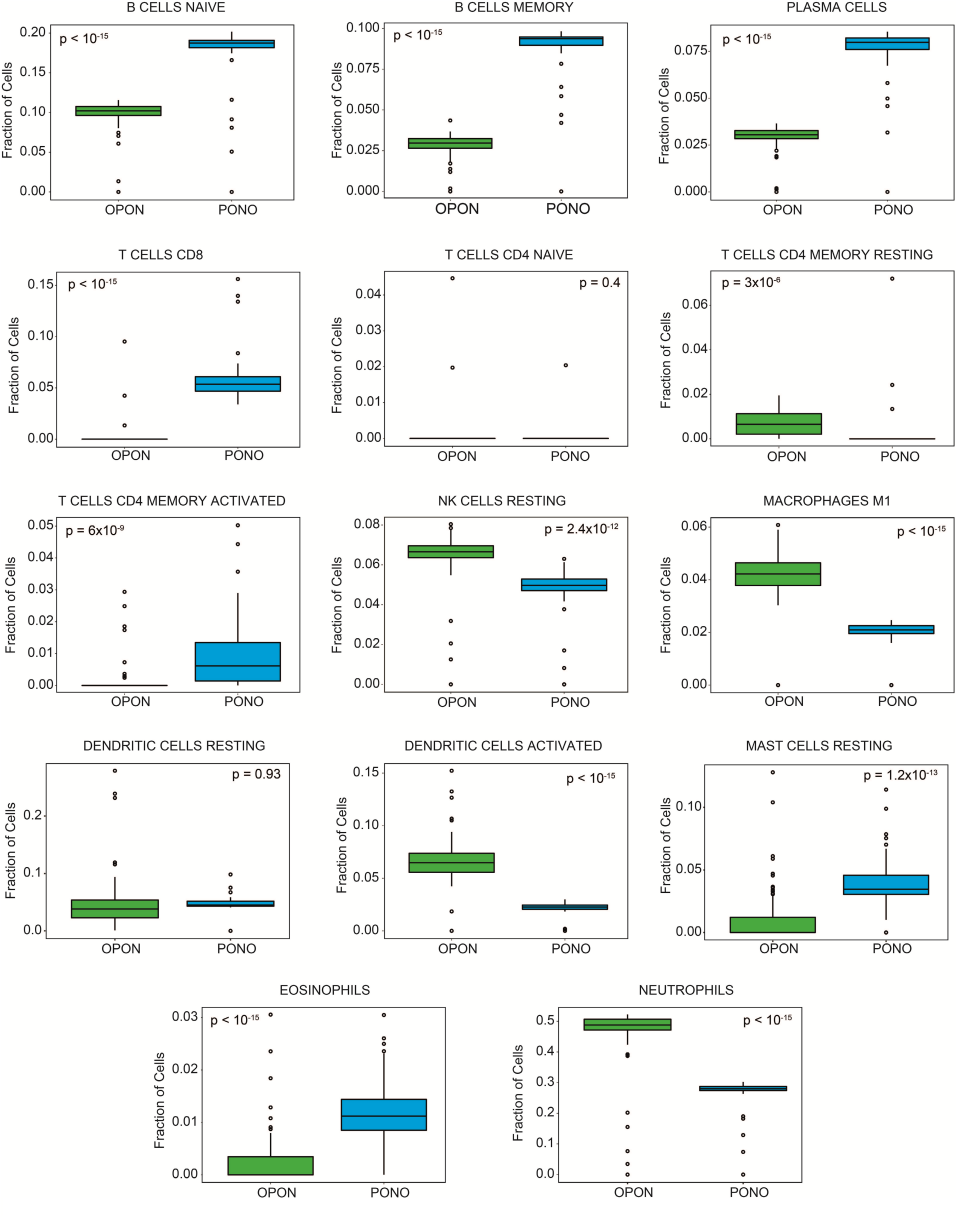
Immune cell specific expressions of hub genes.

Many attempts have been made to redirect the immune system against malignant blasts because it is well known that leukemic blasts acquire the ability to evade immune surveillance and promote disease progression by disrupting their immunological environment.^36^ Therefore, we investigated how the immunological microenvironment alters the treatment response. CIBERSORT algorithm was applied to deconvolute PONO and OPON networks by the custom gene expression signature reference matrix (Figure 4). The results showed that the abundance of CD4 T cells (memory resting and activated) was significantly different in the PONO and OPON networks (Figure 4). Plasma cells, eosinophils, memory cells, and naïve B cells were highly enriched in the PONO network. In addition, M1 macrophages, activated dendritic cells, neutrophils, and activated mast cells were found to be closely associated with immune infiltration in the OPON network.

Tumor morphology is determined by the neoplastic cell component and the immunological microenvironment within tumor cells, which can undermine the host immune response. From this perspective, the tumor environment represents a promising and viable field for the development of new biomarkers. In any case, single cell analysis of AML samples is required to fully understand the importance of hub genes in the tumor microenvironment.

## 4 DISCUSSION

AML is a clinically diverse disease characterized by various chromosomal abnormalities and gene mutations that result in significant differences in response and survival after therapy. Overcoming tumor relapse and drug resistance is a critical obstacle in the treatment of AML. The farnesyltransferase inhibitor tipifarnib has been used to inhibit farnesyltransferase function by blocking prenylation of the CAAX tail motif. It prevents Ras from binding to the membrane and renders it inactive. The Ras signaling pathway is a potential therapeutic target for AML, as many AML patients have mutations in NRAS, KRAS, or genes that stimulate Ras signaling.^37^

The main goal of this study was to build networks around genes based on variations in differential co‐expression patterns of treatment status. It is important to remember that genes involved in complex diseases such as cancer never work alone but interact in a complicated and interconnected system. We used comparative and integrative analysis to create a co‐expression network for drug response and nonresponse. Data from refractory, relapse, and previously untreated AML patients were extracted from datasets and analyzed. Identification of changes in co‐expression patterns of genes in patients could provide information about possible treatment‐related genes and their co‐expressed network for the treated patients who did not respond to previous therapy and/or for the patients who were untreated. The previous studies that provided information on the gene expression ratio and gene expression signature aimed to categorize AML patients based on their likelihood of responding to tipifarnib treatment. These studies primarily focused on identifying specific gene expression assays as potential predictive markers for treatment response. Beyond the expression of individual genes, our study is expected to provide valuable insights into the underlying molecular mechanisms that determine response to tipifarnib. By examining the dynamic interactions and crosstalk between genes, we aim to uncover potential biomarkers and key regulatory nodes within co‐expressed networks that may serve as better predictors of treatment response. Consequently, the unique co‐expressed gene networks uncovered here could be considered as “systems biomarkers” that pave the way to identify patients most likely to respond to and benefit from therapy. Most research that has used methods to analyze differential co‐expression networks has focused primarily on positive correlations and ignored the biological implications of negative correlations.^38^ In this study, negative and positive correlations in a network (PONO for patients who respond to therapy, OPON for patients who do not respond) were examined based on response status to determine how different correlations interact and influence drug response.

The genes in the PONO network were mainly enriched in the MAPK pathway, the cycle of Rho GTPases, and semaphorin interactions. Ras and Rho family GTPases play essential roles in signal transduction during MAPK cascade activation.^39^ In semaphorin interactions, it was discovered that the MAPK family is activated in response to different axon guidance molecules. MAPK activation in semaphorin signaling has been found to be associated with cytoskeletal control.^40^ Semaphorins regulate cytoskeletal structure via modulation of Rho family proteins and cytoskeletal attachment via R‐Ras signaling.^41^ Genes in the OPON network were enriched in alanine and aspartate metabolism, TCR signaling, GABA, and leptin signaling. Leptin receptors were highly expressed in AML cell lines and contributed to proliferation. Moreover, the receptors were constitutively expressed in primary leukemic cells, newly diagnosed, refractory, and relapsed AML patients.^42^, ^43^

The topological metrics of the networks were considered to determine a highly associated gene group in the AML based on response and nonresponse status and to define hub gene‐related modules. SECISBP2L (selenocysteine incorporation),^44^, ^45^ MAN1A2 (metabolic process),^46^, ^47^, ^48^, ^49^ PRPF31 (mRNA splicing),^50^, ^51^ VASP (cell junction assembly),^52^ and SNAPC1 (transcription regulation)^53^, ^54^ were identified as highly connected gene groups in the PONO network. Moreover, PHTF2 (transcription regulation),^55^ SLC11A2 (heme process),^56^ PDLIM5 (cell–cell adhesion),^57^, ^58^, ^59^ OTUB1 (adaptive immune system),^60^, ^61^, ^62^, ^63^, ^64^ and KLRD1 (immune response regulation)^65^, ^66^ were found in the OPON network (Table 2).

**TABLE 2:** The biological meaning and descriptions of network related Hub genes proposed in the current study.

Gene List	Name	Functioning in human diseases
SECISBP2L	SECIS Binding Protein 2 Like	It is highly expressed in the central nervous system^44^ and was suppressed in lung cancer, slows down the cell proliferation^45^
MAN1A2	Mannosidase Alpha Class 1A Member 2	It has key role in cancer and immune‐related activity such as inflammation and pathogen infection^46^, ^47^ and was identified as a prognostic factor for B cell lymphoma^48^ and the participation of α‐1,2 mannosidases in cancer has been described, its function remains unknown.^49^
PRPF31	Pre‐mrnas Processing Factor 31	It has been associated with Retinitis Pigmentosa (RP)^50^ and its genetic variants have been found as worse metformin response in type 2 diabetes.^51^
VASP	Vasodilator‐Stimulated Phosphoprotein	Phosphorylation of VASP on Ser157 was recovered in Imatinib‐responsive individuals but not in those who were resistant.^52^
SNAPC1	Small Nuclear RNA Activating Complex Polypeptide 1	It can interact with Rb and p53, suggesting that it may play a role during the cell cycle^53^ and has been associated to breast cancer tumorigenesis.^54^
PHTF2	Putative Homeodomain Transcription Factor 2	It was reported that fatty acid metabolism mediated by PHTF2 can greatly impact the tumorigenic potential of gastric cancer cells both in vivo and vitro studies.^55^
SLC11A2	Solute Carrier Family 11 Member 2	It was associated in iron uptake as a transmembrane iron transporter, plays crucial roles in transferrin cycle endosomes in erythroid precursors, hepatic iron overload, iron absorption in the intestinal system, and many other processes.^56^
PDLIM5	PDZ and LIM Domain 5	The upregulation of PDLIM5 was reported in many cancer types, such as prostate,^57^ and papillary thyroid carcinoma^58^ and was associated with poor outcomes in lung cancer, and its existence in lung tissue causes cell adhesion, migration, invasion, and metastasis.^59^
OTUB1	OTU domain‐containing ubiquitin aldehyde‐binding protein 1	It functions critically in the DNA damage response, cell apoptosis, proliferation, and cancer development. It stimulates the activation of RAS in lung cancer,^60^ plays a role in deubiquitination and stabilization of FOXM1 in ovarian^61^ and breast cancer,^62^ promotes invasion in the colon^63^ and activates RhoA‐mediated invasion in prostate cancer.^64^
KLRD1	Killer Cell Lectin Like Receptor D1	The low expression and the inhibiting and activating form was detected in AML patients compared to healthy subjects.^65^ The higher expression of neural cell adhesion molecule (NCAM) and CD94 was associated with early death in pediatric AML.^66^

SECISBP2L plays a role in selenoprotein expression but does not promote Sec incorporation in vitro.^67^ It is likely the result of a whole‐genome duplication early in the vertebrate lineage.^67^ Discovery of the function of this protein is still in the early stages. MAN1A2 encodes a protein belonging to a mannosyl‐oligosaccharide family and functions in the Asn‐linked oligosaccharides' maturation in the Golgi complex.^47^ Members of this family play key roles in the folding, maturation, and transport of glycoproteins and are involved in cancer and immunological activities such as inflammation and infection with pathogens.^46^, ^47^ PRPF31 encodes a part of spliceosome complex, is essential for cell metabolism and survival. The study showed that PRPF31 protein plays a direct role in the mitotic process and is associated with spindle microtubules.^68^ VASP has a proline‐rich domain that binds SH3 and WW domain‐containing proteins. Zyxin and VASP are associating proteins that have roles in cellular adhesion and movement. Their involvement and interaction of VASP and Zyxin were studied in K562 cells, and their inhibition caused to decrease in the expression of anti‐apoptotic proteins such as BCL‐XL and BCL2.^52^ SNAPC1 functions as a basal transcription factor to promote snRNA transcription. Beyond snRNA genes, SNAPC1 chromatin occupancy included many transcriptionally active protein‐coding genes.^53^

PHTF2 is an evolutionarily conserved gene expressed primarily in muscle tissue^69^ and has been associated with mediating stress transcription. Although its essential paralog PHTF1 has been overexpressed in T‐ ALL cell lines to regulate cell proliferation and apoptosis,^70^ the association of PHTF2 with other hematological cancers has not been reported. SLC11A2 is associated with iron uptake as a transmembrane iron transporter and plays a critical role in transferrin cycle endosomes in erythroid progenitor cells.^56^ AML patients have been found to have higher serum ferritin at diagnosis, which stimulates leukemia cell development while suppressing normal colony formation of progenitor cells.^71^, ^72^ Moreover, in patients with hematologic malignancies after allogeneic hematopoietic stem cell transplantation, an elevated serum ferritin level is a poor prognostic indicator of overall survival and non‐relapse mortality.^73^ Although direct associations between SLC11A2 and AML prognosis have not been reported, there may be a causal effect of dysregulation of SLC11A2 on AML prognosis. PDLIM5 functions in cell proliferation and differentiation in many cell types and tissues. It was identified as a protein kinase that binds to PKC, PKD, AMPK, and PKA. The study showed that the expression of PDLIM4 was lower in AML patients than in healthy controls. The lower PDLIM4 expression resulted in longer overall survival than AML patients without PDLIM4.^74^ Another study claimed that high expression of PDLIM2 and PDLIM7 are negative prognostic factors in AML patients, but their influence on survival was not found in allo‐HSCT recipients.^75^ Although the interactions and functions of various PDLIM family members have been demonstrated in AML studies, the association of PDLIM5 has not yet been reported.

OTUB1 is characterized by an ovarian tumor (OTU) domain and encodes a protein that is a specific ubiquitin iso‐peptidase that cleaves ubiquitin from branched poly‐ubiquitin chains. OTUB1 may play a critical role in cancer treatment because cancer cells rely on a functioning ubiquitin‐proteasome system (UPS), making it an attractive target for developing new therapies with selectivity for tumor cells. KLRD1 encodes an antigen (CD94) expressed mainly on natural killer (NK) cells. It forms a heterodimer with NKG2A (inhibitory form), NKG2C, or NKG2E (activating form) and binds to the non‐classical MHC I molecule and HLA‐E in humans. Expression of HLA‐E in tumors suppresses NK cell function via the inhibitory receptor CD94/NKG2A on the surface of NK cells, resulting in immune cell resistance to tumor.^76^ After treatment of AML patients with haplo‐mismatched SCT, NK cells were rapidly generated, and higher expression of CD94/NKG2A was detected in the NK cells than in healthy donors.^77^ Moreover, uncovering other possible KLRD1(CD94) interactions in AML blast cells may reveal predisposing factors for disease prognosis and treatment resistance.

Transcriptional regulators of genes were examined, and five TFs, ETS1, GATA2, AR, YBX1, and FOXP3, were found to be significant in both networks. ETS1 expression has been found to be increased in childhood AML; however, the functional implications of ETS1 overexpression in AML are not yet known.^78^ GATA2 plays a critical role in hematopoietic stem and progenitor cell formation, and overexpression is associated with poor prognosis in AML patients.^79^ Hu et al. demonstrated the comprehensive pan‐cancer analysis of AR in various tumor types and found that AML patients with high AR expression had a higher survival rate.^80^ FOXP3 is critical for the development or function of Tregs; its expression was increased in newly diagnosed and relapsed/refractory patients.^81^ The recent study showed that YBX1 is a particular dependency and therapeutic target in AML, required for oncogenic protein production.^82^

The malignancy of various cancers has been associated with the presence of tumor‐infiltrating immune cells. Determining the status of the immune response in individual patients at each stage of the disease is critical for deciding subsequent clinical diagnosis and treatment. On the other hand, immune profiling offers the potential to identify biomarkers of response to immunotherapy, providing important information to improve clinical trial design by using techniques to modify anti‐leukemia immunity for AML treatment. Several clinical studies have shown that T cell immunity is impaired in AML in multiple ways, including an increase in T‐regulatory cells and a decrease in T helper cells, T cell exhaustion, and abnormal transcription factor activity.^83^, ^84^ AML patients have impaired immunological responses due to immunosuppressive circuits activated by soluble factors and checkpoint molecules, including PD‐L1, TIM‐3, and IDO‐1. CD8+ T lymphocytes with increased PD‐1 expression may contribute to cytotoxic T cell dysfunction and immune response suppression in patients with advanced AML.

B cells can mature into plasma cells that generate antibodies, but they can also perform several additional roles that help the immune system eliminate tumor cells. The number of CD19+ B cells has been shown to decrease substantially and significantly in relapsed and refractory AML patients compared to healthy donors.^85^ Another study indicated that the number of B cells recovered in patients who fully responded to AML treatment, demonstrating that B cells may play a role in the development of AML.^86^ Survival analysis performed in the study showed that mast cell quiescence had a significant association with survival of AML patients.^87^ Nevertheless, patients with AML may benefit from treatment that targets their mast cells. Macrophages may play a role in treatment resistance in AML, but the role in the overall mechanism is still unclear. Interaction between macrophages and AML cells and/or substances secreted by macrophages may reduce AML cell sensitivity to drugs, and macrophages may be retrained by AML blasts to support leukemia, according to preliminary findings.^88^ Recent research by Al‐Matary et al. has greatly improved our knowledge of how leukemia‐associated macrophages (LAMs) protect AML cells.^89^ They discovered that AML cells were responsible for the invasion of LAMs into the bone marrow and spleen of leukemia patients as well as mice. LAMs supported in vitro development of AML cell lines better than macrophages from non‐leukemic mice in various gene mutation‐induced AML mouse models. LAM infiltration was found to be correlated with mouse survival.

Antigens are presented by dendritic cells (DCs) that trigger specific T cell responses. They are also responsible for the recruitment and activation of NK cells and the differentiation of naive T cells. Tumor‐forming plasmacytoid DCs (TF‐PDCs) from AML patients have been shown to express leukemia‐associated proteins in several studies. The study showed that patients with TF‐PDCs had median lower sensitivity to standard chemotherapy regimens and resistance to pirarubicin and sorafenib.^90^ They suggested that TF‐PDC positive AML patients may have primary chemotherapy resistance and TF‐PDC in the AML microenvironment may be responsible for drug resistance and disease relapse. Within the scope of that research, the overall survival of TF‐PDC positive AML patients was found to be shorter than that of TF‐PDC negative patients.^90^ Although neutrophils have an anti‐inflammatory effect, they also secrete cytokines that promote cancer progression, such as interleukin and tumor necrosis factor α.

On the other hand, lymphocytes are crucial components of the immune system in fighting cancer cells. Recent studies have shown that neutrophil to lymphocyte ratio (NLR) is a biomarker of the patient's immune response in the tumor microenvironment.^91^ Zhang et al. indicated that AML patients with a high NLR may have a decreased antitumor response, leading to an increased risk of death. In addition, Mushtaq et al. showed that a high NLR in relapsed/refractory AML patients indicates a poor prognosis.^92^

The clear delineation of these subgroups helps to better interpret the results and understand the impact of cytogenetic risk on treatment outcomes in AML, which contributes to the further development of tailored therapeutic approaches. Further research and analysis in this context may help elucidate the underlying mechanisms and develop strategies to improve treatment efficacy in specific AML subtypes. It is essential to clarify whether the identified findings truly encompass any specific AML subtype, considering the inherent heterogeneity of the disease. Moreover, we recognize the importance of validating our assessment model with clinical samples but must regret that constraints and limitations did not allow such experiments to be performed in this study. Nevertheless, we ensured rigorous analysis and used publicly available transcriptomic data to provide valuable insights for future investigations in this area.

We expect to gain valuable insights into how the tumor environment may influence host immune responses and treatment outcomes. By studying co‐expression networks involving immune cells, biomarkers and therapeutic targets for AML could be identified, considering the impact of tumor morphology, which is influenced by both neoplastic cell components and the immunological microenvironment. Despite recognizing the need for further investigation and validation is needed, such as single‐cell transcriptomics, our emphasis on analyzing the tumor microenvironment contributes to our understanding of the complexity of AML and paves the way for innovative treatment approaches. Acknowledging and addressing these limitations will improve the interpretation and generalizability of the results of our study and provide a more comprehensive understanding of the impact on different AML subgroups, using clinical samples and providing a foundation for future studies aimed at refining therapeutic approaches based on subtype specific.

In this study, our main focus was not to analyze the data based on specific AML subtypes, but rather to understand the response mechanisms derived from the overall results. The goal was to gain insight into treatment response across the entire cohort, independent of cytogenetic subgroups. By examining the overall data, we aimed to identify common trends and factors influencing treatment outcomes in the patient population studied. This approach allowed us to explore potential therapeutic targets and develop broader strategies that might be applicable to different AML subtypes. Although we recognize the importance of studying specific subtypes in future research, our current study should provide valuable information on mechanisms of response to treatment in AML and provide a basis for more focused investigations in subsequent studies.

This study allows us to shed light on the prognosis of AML based on response status. In any case, more needs to be done to confirm this clinically, and the relevant underlying process is still being elucidated. We identified the potential prognostic biomarkers in AML based on the treatment responses of patient data on gene co‐expression networks. Two networks were identified based on response and were found to be predictive for both AML and CLL. As a result, novel genes were identified as highly connected in the networks and may be associated with patient response to therapy.

The application of targeted therapy in AML has been difficult because of the complexity of mutational processes within and between individuals and the lack of pharmacological drugs for most mutational events. Identifying therapeutically relevant pharmacogenomic interactions by determining gene‐drug interactions is a powerful method. By applying bioinformatics approaches, we have constructed co‐expression networks that can be used to clinically assess drug response and classify patients who respond. It should be remembered that genes associated with cancer function together in the complex networks of cells. From this point of view, in our study, we looked at negative and positive correlations in a response status‐ based network to see how different correlations interact and influence drug response. SECISBP2L, MAN1A2, PRPF31, VASP, SNAPC1 in the response network and PHTF2, PDLIM5 and OTUB1 in the nonresponse network were proposed as novel network biomarkers.

## AUTHOR CONTRIBUTIONS

**Nurdan Kelesoglu:** Conceptualization (equal); formal analysis (equal); writing – original draft (lead). **Medi Kori:** Formal analysis (supporting); writing – original draft (supporting). **Betul Yilmaz Karademir:** Supervision (equal); writing – review and editing (supporting). **Ozlem Ates Duru:** Conceptualization (equal); writing – original draft (supporting). **Kazim Yalcin Arga:** Conceptualization (lead); writing – original draft (lead); writing – review and editing (lead).

## FUNDING INFORMATION

The present study was enabled by a research grant from the Health Institutes of Turkey (TUSEB) with grant number 2019‐TA01‐4065.

## CONFLICT OF INTEREST STATEMENT

The authors declare no conflicts of interest regarding this study.

## ETHICS STATEMENT

The current study is exempt from ethical approval according to national legislation, since publicly available datasets were employed.

## CONSENT

The current study is exempt from new consents from patients according to national legislation, since written informed consents were declared by the original publications reporting the publicly available datasets.

## Supporting information


**Figure S1.** Biological process and pathway enrichments of (A) upregulated and (B) downregulated genes.

**Figure S2.** Co‐expressed hub genes module of response network.

**Figure S3.** Co‐expressed hub genes module of nonresponse network.

## Data Availability

Publicly available datasets were analyzed in this study. The datasets analyzed during the current study are available in GEO and The Genome Cancer Atlas (https://www.ncbi.nlm.nih.gov/geo/ and https://portal.gdc.cancer.gov/). The original contributions presented in the study are included in the article/Supplementary Material. Further inquiries can be directed to the corresponding author.
