# Supplementary figures and images for: Differential co‐expression network analysis elucidated genes associated with sensitivity to farnesyltransferase inhibitor and prognosis of acute myeloid leukemia
Source: Cancer Med. 2023 Dec 8;12(24):22420–36. doi: 10.1002/cam4.6804 (PMC10757125; doi:10.1002/cam4.6804)

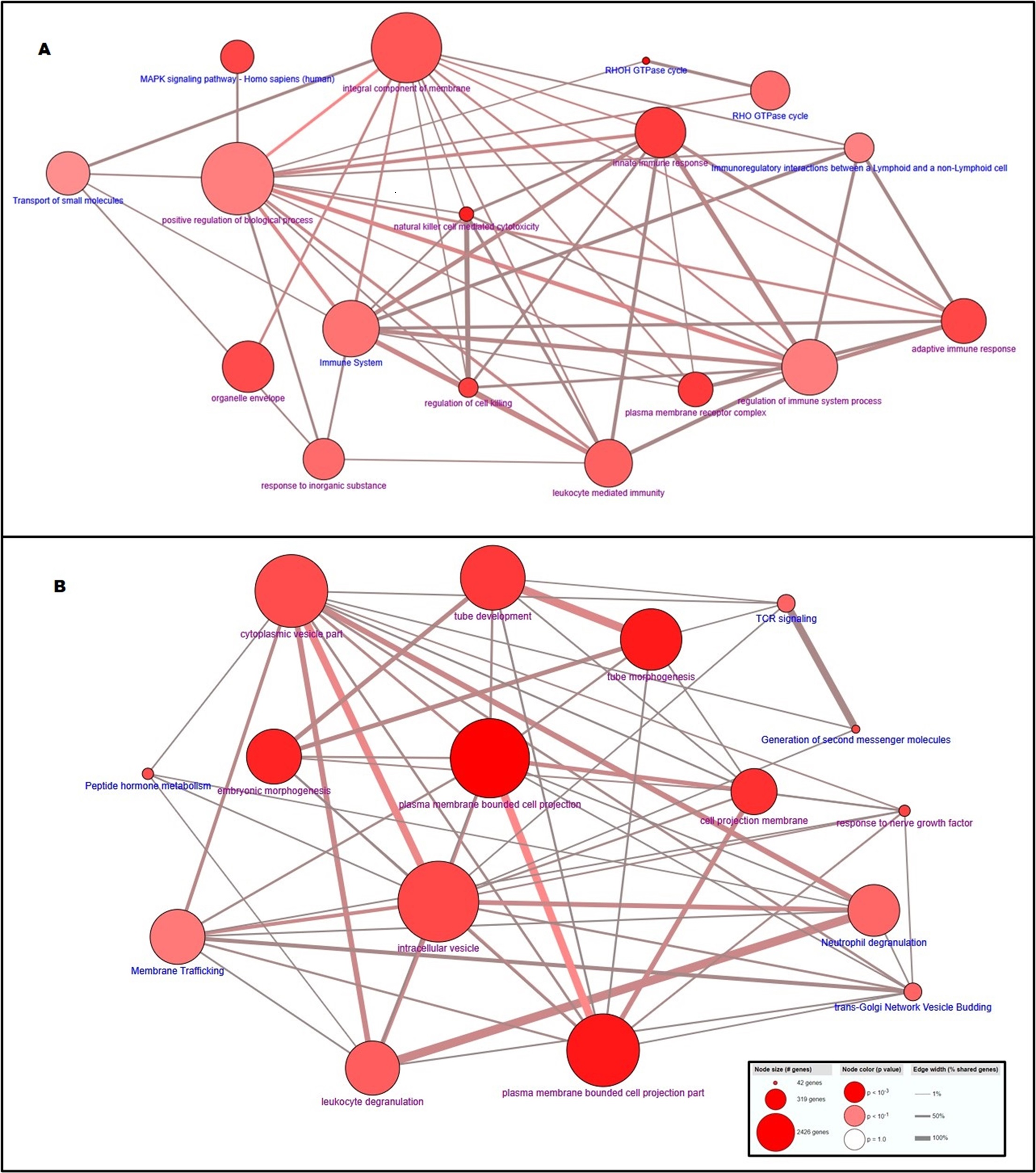

Supplement: Supplementary file 1 — Figure S1. Biological process and pathway enrichments of (A) upregulated and (B) downregulated genes. [file CAM4-12-22420-s002.jpg]

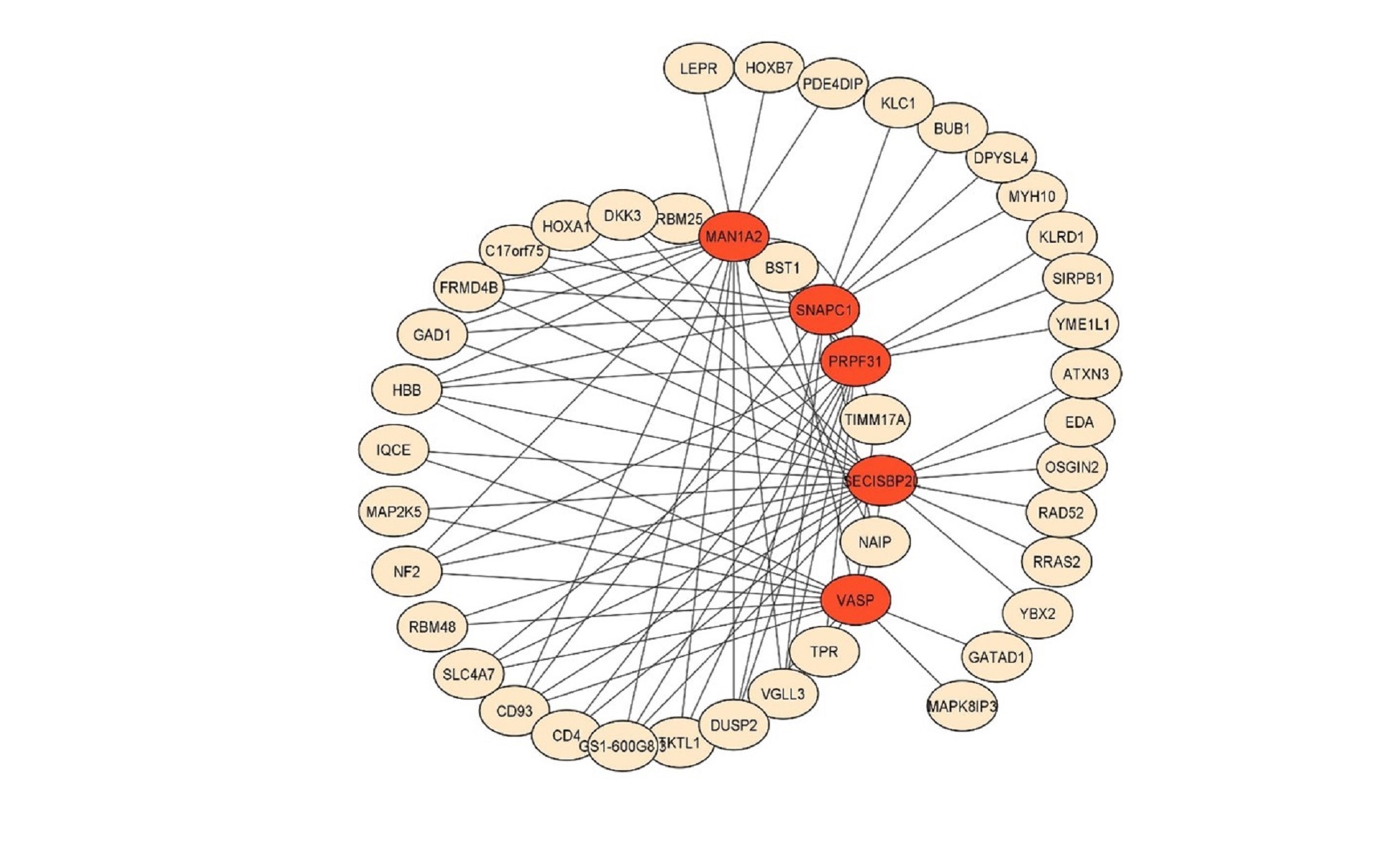

Supplement: Supplementary file 2 — Figure S2. Co‐expressed hub genes module of response network. [file CAM4-12-22420-s001.jpg]

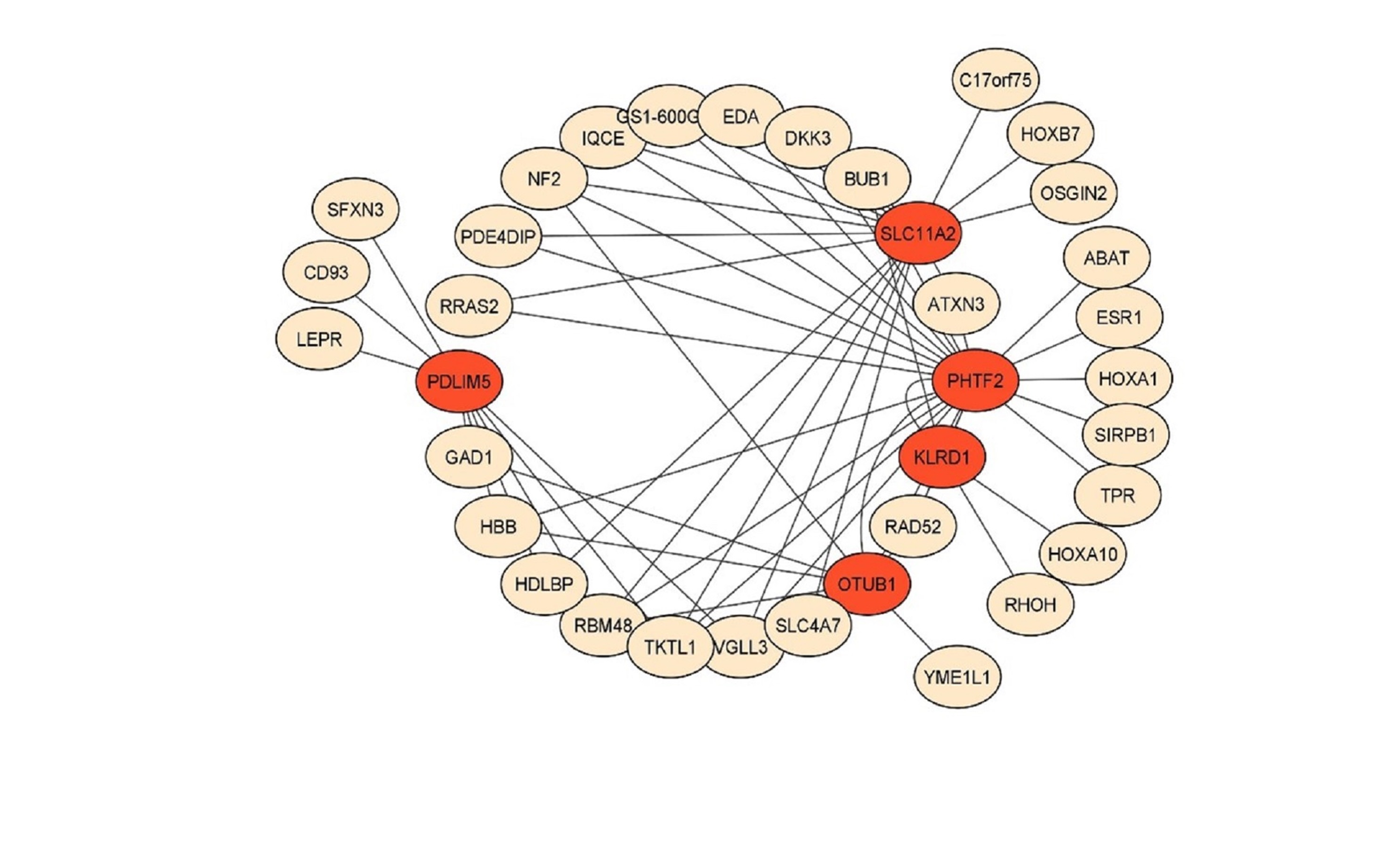

Supplement: Supplementary file 3 — Figure S3. Co‐expressed hub genes module of nonresponse network. [file CAM4-12-22420-s003.jpg]
